# Current advances on the phytochemistry, pharmacology, quality control and applications of *Anoectochilus roxburghii*

**DOI:** 10.3389/fphar.2024.1527341

**Published:** 2025-01-03

**Authors:** Xiaoxue Zou, Kexin Zhang, Xuezhen Li, Yuqin Zhang, Lixia Chen, Hua Li

**Affiliations:** ^1^ Fujian Key Laboratory of Chinese Materia Medica, College of Pharmacy, Institute of Structural Pharmacology and TCM Chemical Biology, Fujian University of Traditional Chinese Medicine, Fuzhou, China; ^2^ Key Laboratory of Structure-Based Drug Design and Discovery, Ministry of Education, Wuya College of Innovation, School of Pharmacy, Shenyang Pharmaceutical University, Shenyang, Liaoning, China

**Keywords:** *Anoectochilus roxburghii* (Wall.) Lindl., phytochemistry, pharmacology, quality control, medicinal food

## Abstract

*Anoectochilus roxburghii* (Wall.) Lindl. (AR) is a perennial herb that has long been used as medicinal and edible plant. In Traditional Chinese Medicine (TCM), AR is utilized to treat various diseases including hyperuricemia, type 2 diabetes mellitus, cancers and inflammatory diseases. Recent advances in the discovery and isolation of bio-active compounds have unveils the main medicinal ingredients, such as quercetin, kinsenoside and rhamnazin. Pharmacological studies further demonstrated its activities, containing anti-inflammation, anti-oxidation, and antihyperlipidemia effects. The processed AR products have various commercial applications in functional foods and cosmetics. AR has been used to prepare soup, drinkbeverage, jelly, face masks, soap, etc. However, despite the abundant medicinal value, it hasn't been included in the 2020 Chinese Pharmacopoeia up to now. There is also no consistent evaluation standard across provinces. This seriously affects the safety and the efficacy of TCM prescriptions, not to mention the development question. This review summarizes recent research on AR in phytochemistry, pharmacology, quality control and applications, raises the corresponding solutions to provide references and potential directions for further studies.

## 1 Introduction

*Anoectochilus roxburghii* (Wall.) Lindl. (AR) is a perennial herb belonging to the genus *Anoectochilus* in the Orchidaceae family, which is distributed widely in subtropical regions. Especially in the southern provinces of China, such as Zhejiang, Fujian and Taiwan. As a tonic with high value, AR is also a well-known supplement throughout the world. Thus, this massively valuable herb ensures high economic efficiency in market. Processed products have multifarious commercial applications in health products, foods and cosmetics (Ye et al., 2017). It is frequently used in food supplements and nutritional products, anti-aging and whitening cosmetics, and even as a raw material for fermentation and winemaking. Thus, it is worthwhile to investigate the immense potential and wide-ranging development possibilities of AR.

Apart from the dietary value, the whole plant of AR is clinically applied in the treatment of hyperuricemia, type 2 diabetes mellitus, cancers, acute and chronic hepatitis, as well as other inflammatory diseases (Ye et al., 2017). To date, pharmacological studies have demonstrated the broad effects of AR, including anti-inflammation (Deng et al., 2022), anti-oxidation (Thong et al., 2019), antitumor (Mao et al., 2022), anti-viral (Zhou, 2017) and anti-hyperlipidemia (Tian et al., 2022). Meanwhile, phytochemical studies of AR identified various bio-active compounds, such as flavonoids, polysaccharides, glycoside, alkaloids, volatile oil, steroids and triterpenes (Xiao et al., 2018). AR has been widely acknowledged as a highly valuable botanical specimen in medication, owing to its remarkable therapeutic efficacy. In the past few decades, the year-over-year increase in market demand for AR resulted in large-scale tissue culture. However, a standardized production and evaluation framework for AR is abscent, as it is not included in the 2020 edition of the Chinese Pharmacopoeia or the Provincial Standard of Chinese Medicinal Materials. Thus, this vacancy tremendously restricted the application of AR in medical and dietary domain, and also largely hindered its pharmacological research. This review focused on the advancements in the study of phytochemistry, pharmacology, quality control and applications of AR, while at the same time, providing scientific evidence for its development.

## 2 Phytochemistry

**FIGURE 1:**
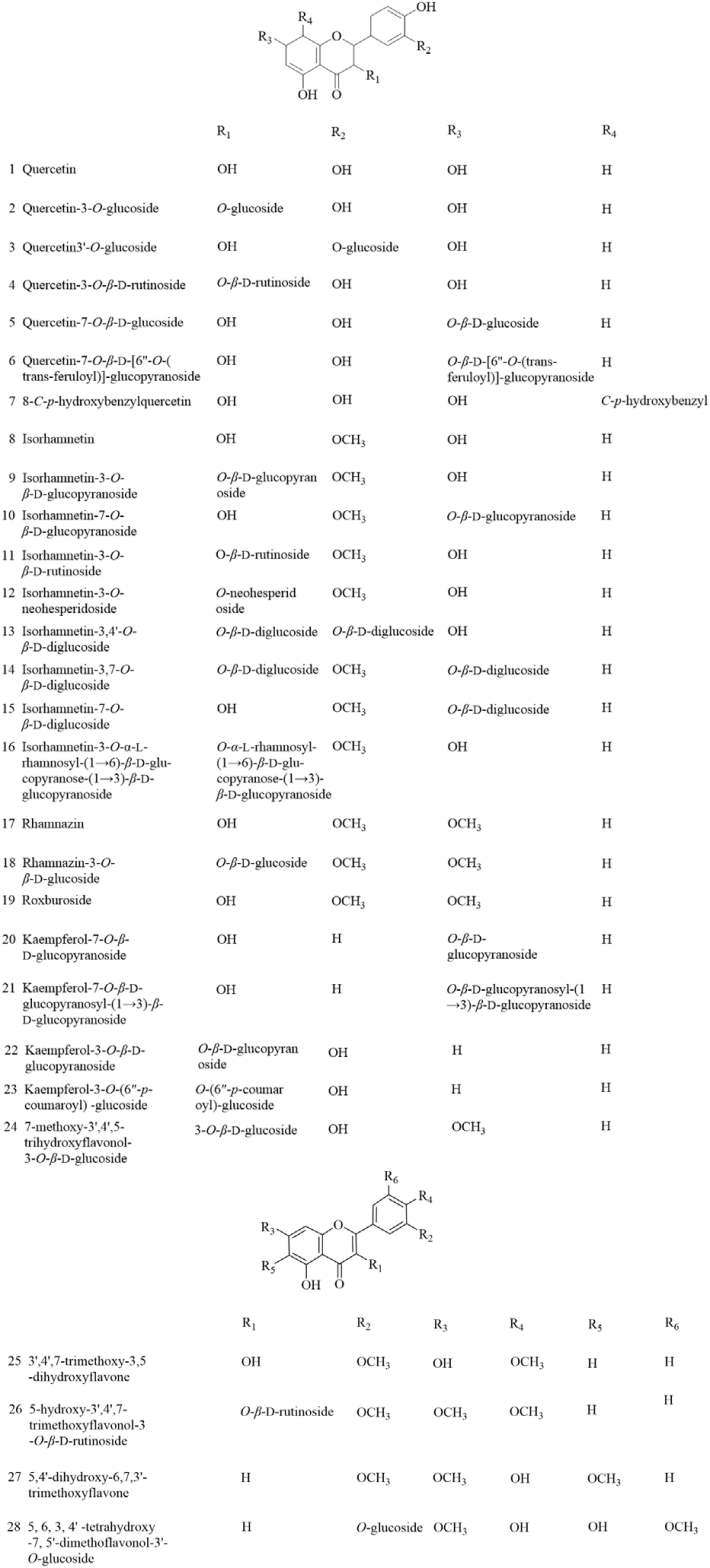
Flavonoids and flavonoid glycosides isolated from AR.

**FIGURE 2:**
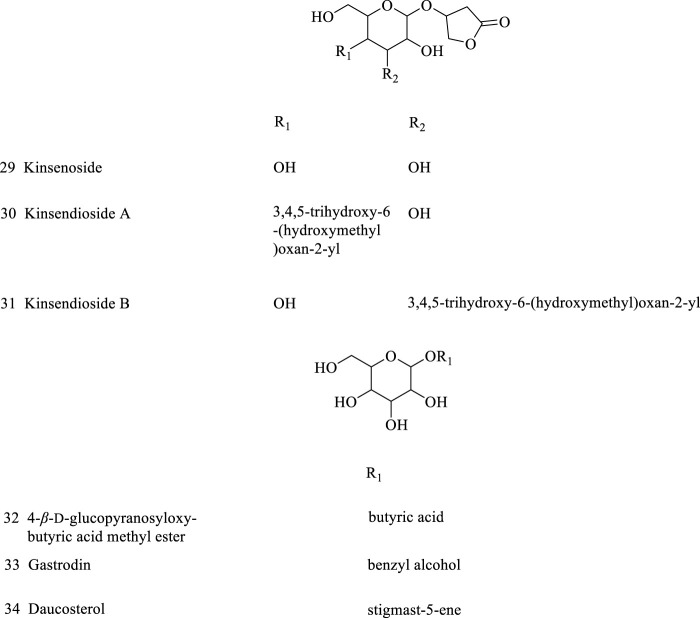
Other glycosides isolated from AR.

**FIGURE 3:**
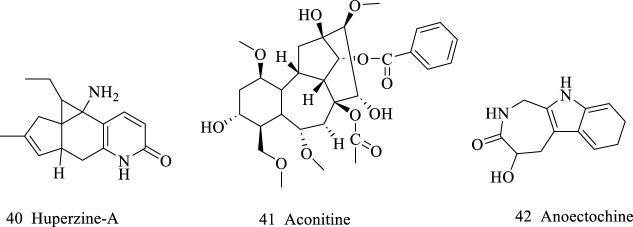
Alkaloids isolated from AR.

**FIGURE 4:**
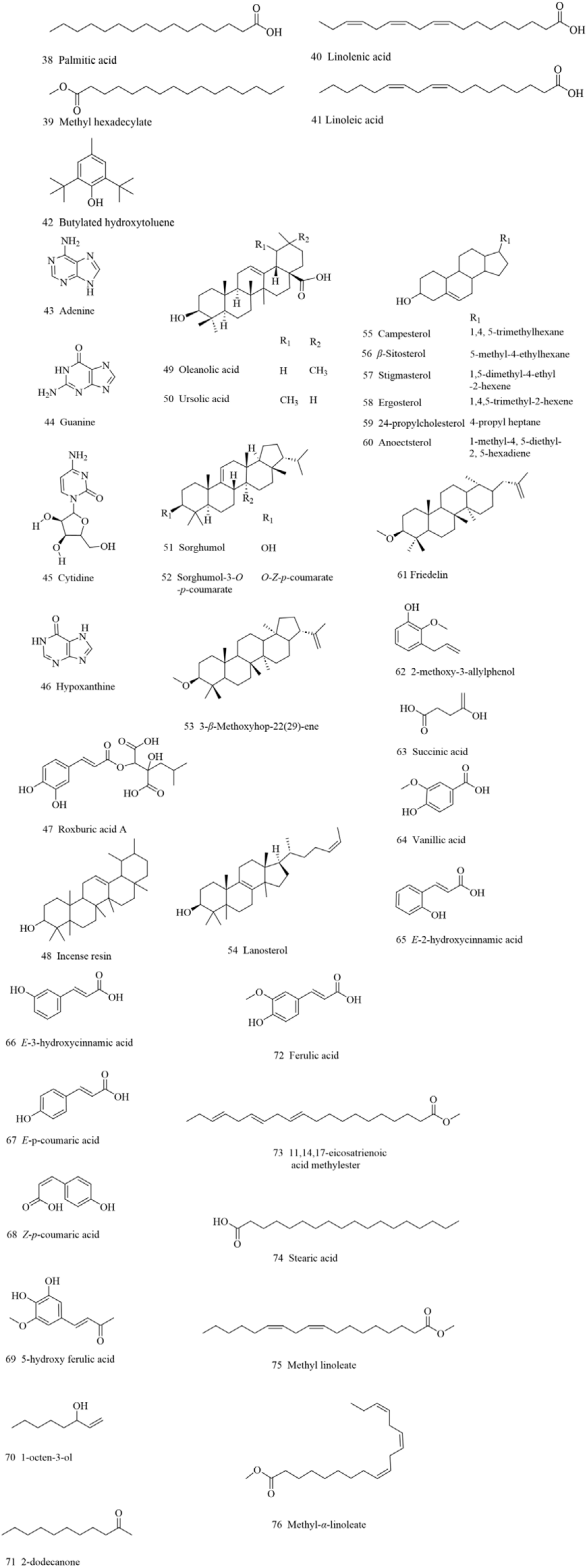
Other compositions isolated from AR.

**FIGURE 5:**
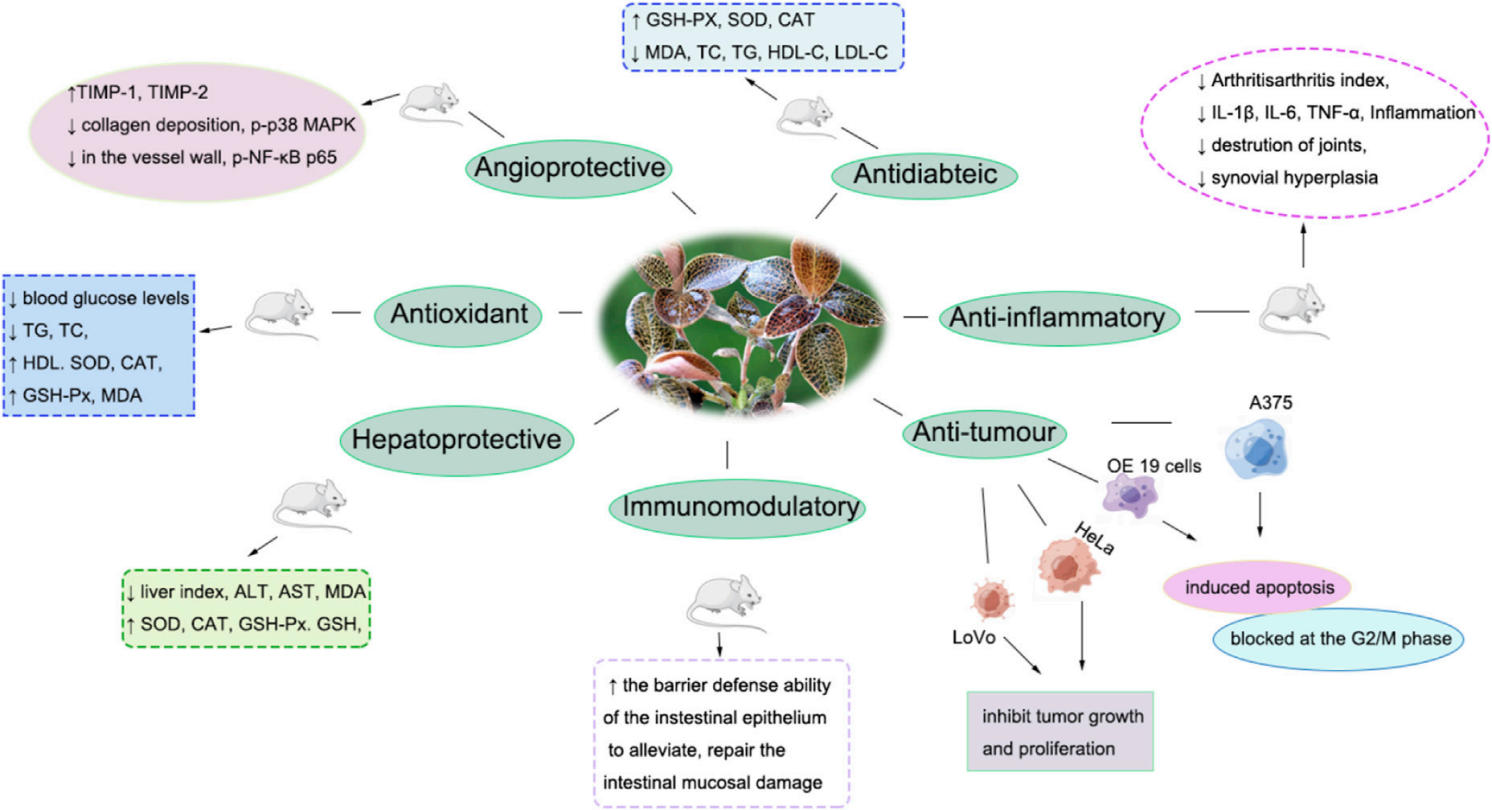
The pharmacological effects of AR.

The chemical constituents of AR are complex, with more than 76 kinds of substances with diverse structures have been identified. Among them, flavonoids, polysaccharides, glycosides, alkaloids, volatile oils, triterpenes, steroids, and trace elements are main pharmacological with pharmacological properties. Tables 1, 2 provide a summary of the chemical composition of AR, and their corresponding structures are depicted in Figures 1–5.

**TABLE 1:** Chemical compounds derived from AR.

Number	Chemical composition	Molecular formula	Reference	Molecular weight (g/mol)	CAS number
Flavonoids and flavonoid glycosides					
1	Quercetin	C_15_H_10_O_7_	Chen J. H. et al. (2023)	302.24	117-39-5
2	Quercetin-3-O-glucoside	C_21_H_20_O_12_	He et al. (2005a)	464.383	21637-25-2
3	Quercetin-3′-O-glucoside	C_21_H_20_O_12_	He et al. (2005a)	464.383	19254-30-9
4	Quercetin-3-O-β-D-rutinoside	C_27_H_30_O_16_	He et al. (2005c)	610.518	949926-49-2
5	Quercetin-7-O-β-D-glucoside	C_21_H_20_O_12_	He et al. (2005c)	464.376	491-50-9
6	Quercetin-7-O-β-D-[6″-Ο-(trans-feruloyl)]-glucopyranoside	C_31_H_28_O_15_	Guan et al. (2005)		
7	8-C-p-hydroxybenzylquercetin	C_22_H_16_O_8_	He et al. (2005a) Guan et al. (2005)		
8	Isorhamnetin	C_16_H_12_O_7_	He et al. (2005a)	316.26	480-19-3
9	Isorhamnetin-3-O-β-D-glucopyranoside	C_22_H_22_O_12_	Guan et al. (2005)	478.4	5041-82-7
10	Isorhamnetin-7-O-β-D-glucopyranoside	C_22_H_22_O_12_	Guan et al. (2005)	478.41	6743-96-0
11	Isorhamnetin-3-O-β-D-rutinoside	C_28_H_32_O_16_	Guan et al. (2005) Yin et al. (2016)	624.54	604-80-8
12	Isorhamnetin-3-O-neohesperidoside	C_27_H_30_O_16_	Yang et al. (2007)	624.544	55033-90-4
13	Isorhamnetin-3,4′-O-β-D-diglucoside	C_27_H_30_O_17_	He et al. (2005c)	640.543	28288-98-4
14	Isorhamnetin-3,7-O-β-D-diglucoside	C_28_H_32_O_17_	He et al. (2005c)	640.54	6758-51-6
15	Isorhamnetin-7-O-β-D-diglucoside	C_28_H_32_O_1_	He et al. (2005c)	478.40	6743-96-0
16	Isorhamnetin-3-O-a-L-rhamnosyl-(1→6)-β-D-glu-copyranose-(1→3)-β-D-glucopyranoside	C_34_H_42_O_21_	Bin et al. (2023)		
17	Rhamnazin	C_17_H_14_O_7_	Yin et al. (2016)	330.29	552-54-5
18	Rhamnazin-3-O-β-D-glucoside	C_23_H_24_O_12_	Yin et al. (2016)	784.71	52801-24-8
19	Roxburoside	C_31_H_28_O_14_	Xu (2008)		
20	Kaempferol-7-O-β-D-glucopyranoside	C_21_H_20_O_11_	Guan et al. (2005)	448.38	16290-07-6
21	Kaempferol-7-O-β-D-glucopyr anosyl-(1→3)-β-D-glucopyranoside	C_27_H_30_O_16_	Bin et al. (2023)		
22	Kaempferol-3-O-β-D-glucopyranoside	C_21_H_20_O_11_	Guan et al. (2005)	448.38	480-10-4
23	Kaempferol-3-O-(6″-p-coumaroyl) -glucoside	C_30_H_26_O_13_	Yang et al. (2007)	594.52	163956-16-9
24	7-methoxy-3′,4′,5-trihydroxyflavonol-3-O-β-D-glucoside	C_22_H_22_O_12_	Xu (2008)		
25	3′,4′,7-trimethoxy-3,5-dihydroxyflavone	C_18_H_16_O_7_	He et al. (2006)		
26	hydroxy-3′,4′,7-trimethoxyflavonol-3-O-β-D-rutinoside	C_30_H_36_O_16_	Guan et al. (2005)		
27	5,4′-dihydroxy-6,7,3′-trimethoxyflavone	C_18_H_16_O_7_	Shi et al. (2016)	344.32	41365-32-6
28	5,6,3,4′-tetrahydroxy-7,5′-dimethoflavonol-3′-O-glucoside	C_23_H_24_O_13_	Liu et al. (2014)		
Other glycosides					
29	Kinsenoside	C_11_H_20_O_8_	Wang et al. (2011)	264.23	151870-74-5
30	Kinsendioside A	C_16_H_26_O_13_	Cai et al. (2008)		
31	Kinsendioside B	C_16_H_26_O_13_	Xu (2008)		
32	4-β-D-glucopyranosyloxy-butyric acid methyl ester	C_11_H_20_O_8_	Wang et al. (2011)		
33	4-(β-D-glucopyranosyloxy) benzyl alcohol (gastrodin)	C_13_H_18_O_7_	Cai et al. (2008)	286.28	62499-27-8
34	Daucosterol	C_35_H_60_O_6_	Wang et al. (2011) Shi et al. (2016) He et al. (2006)	576.86	474-58-8
Alkaloids					
35	Huperzine-A	C_15_H_18_N_2_O	Han et al. (2008)	242.32	
36	Aconitine	C_34_H_47_NO_11_	Wu et al. (2014)	645.74	302-27-2
37	Anoectochine	C_12_H_12_N_2_O_2_	Han et al. (2008) Wu et al. (2014)		
Other components					
38	Palmitic acid	C_16_H_32_O_2_	Wang et al. (2011) Ito et al. (1993)	256.42	57-10-3
39	Methyl hexadecylate	C_17_H_34_O_2_	Gong et al. (2014)		
40	Linolenic acid	C_15_H_24_O	Gong et al. (2014)	278.43	463-40-1
41	Linoleic acid	C_18_H_32_O_2_	Ito et al. (1993)	280.45	60-33-3
42	Butylated hydroxytoluLinoleic acidene	C_19_H_32_O_2_	Gong et al. (2014)		
43	Adenine	C_5_H_5_N_5_	He et al. (2005b)	135.13	73-24-5
44	Guanine	C_5_H_5_N_5_O	Zhong et al. (2006)	151.13	73-40-5
45	Cytidine	C_9_H_13_N_3_O_5_	Zhong et al. (2006)	243.22	65-46-3
46	Hypoxanthine	C_5_H_4_N_4_O	Zhong et al. (2006)	136.11	68-94-0
47	Roxburic acid A	C_18_H_22_O_9_	Bin et al. (2023)		
48	Incense resin	C_30_H_50_O	Yin et al. (2016) Yang et al. (2007)		
49	Oleanolic acid	C_30_H_48_O_3_	Wu (2013) Guan et al. (2005)	456.7	508-02-1
50	Ursolic acid	C_30_H_48_O_3_	Wu (2013)	456.7	77-52-1
51	Sorghumol	C_30_H_52_O	Ito et al. (1993)	426.7	90582-44-8
52	Sorghumol-3-O-p-coumarate	C_39_H_58_O_3_	He et al. (2005b)		
53	3-β-Methoxyhop-22 (29)-ene	C_31_H_52_O	Yin et al. (2016)	468.75	76582-61-1
54	Lanosterol	C_30_H_50_O	Yang et al. (2007)	426.73	79-63-0
55	Campesterol	C_28_H_48_O	He et al. (2006)	400.69	474-62-4
56	β-Sitosterol	C_29_H_50_O	Wang et al. (2011)	414.72	83-46-5
57	Stigmasterol	C_29_H_48_O	Yin et al. (2016)	412.7	83-48-7
58	Ergosterol	C_28_H_44_O	Ito et al. (1993)	396.66	57-87-4
59	24-propylcholesterol	C28H_49_O	Huang et al. (2007a)	428.73	64997-52-0
60	Anoectsterol	C_30_H_48_O	He et al. (2005b)		
61	Friedelin	C_30_H_50_O	Ito et al. (1993)	426.72	559-74-0
62	2-methoxy-3-allylphenol	C_10_H_12_O_2_	Chen et al. (2010a)	164.20	1941-12-4
63	Succinic acid	C_4_H_6_O_4_	Wang et al. (2011) He et al. (2005a)	118.09	110-15-6
64	Vanillic acid	C_8_H_8_O_4_	Yang et al. (2007)	168.15	121-34-6
65	E-2-hydroxycinnamic acid	C_9_H_8_O_3_	Liu et al. (2014)	164.15	614-60-8
66	E-3-hydroxycinnamic acid	C_9_H_8_O_3_	Liu et al. (2014)	164.15	614-60-8
67	E-p-coumaric acid	C_9_H_8_O_3_	Yin et al. (2016) Yang et al. (2007)	164.16	501-98-4
68	Z-p-coumaric acid	C_9_H_8_O_3_	Yin et al. (2016) Yang et al. (2007)	164.16	4501-31-9
69	5-hydroxy ferulic acid	C_10_H_10_O_5_	Liu et al. (2014)	210.18	1782-55-4
70	1-octen-3-ol	C_8_H_16_O	Huang et al. (2007a)	128.21	3391-86-4
71	2-dodecanone	C_12_H_24_O	He et al. (2005b)	184.32	6175-49-1
72	Ferulic acid	C_10_H_10_O_4_	Chen Z. X. et al. (2023) He et al. (2006)	194.18	1135-24-6
73	11,14,17-eicosatrienoic acid methylester	C_21_H_36_O_2_	He et al. (2005b)	320.51	55682-88-7
74	Stearic acid	C_18_H_36_O_2_	Wang et al. (2011)	284.48	57-11-4
75	Methyl linoleate	C_19_H_34_O_2_	He et al. (2005b)	294.47	68605-14-1
76	Methyl α-linolenate	C_19_H_32_O_2_	He et al. (2005b)	292.45	301-00-8

**TABLE 2:** Composition and analysis of polysaccharides in AR.

Name	Extraction solvent	Analytical method	Monosaccharide composition	Molecular weight (Da)	Reference
ARPP70a	Water	Anion exchange resin, HPGPC, Phenol-sulphuric acid method, FT-IR analysis, NMR spectroscopy	Glucose, galactose	14.8 × 10^3^	Zhang Y. H. et al. (2021)
ARPP30	95% ethanol	GC–MS, UV, HPLC, Refraction index detector, Size exclution chromatography column	Glucose, mannose	59378	Zeng et al. (2016)
ARPP60	95% ethanol	GC–MS, UV, HPLC, Refraction index detector, Size exclution chromatography column	Glucose, mannose	13377	Zeng et al. (2016)
ARPP80	95% ethanol	GC–MS, UV, HPLC, Refraction index detector, Size exclution chromatography column	Glucose, mannose	106.04 × 10^4^	Zeng et al. (2016)
ARLP-W	Water	HPGPC, HPLC, GC–MS, NMR, Phenol-sulphuric acid method, Cellulose DE-52 chromatography	Mannos, glucose	8.1 × 10^4^	Gao L. et al. (2021)
ARPs-p	Water	GC, UV, HPGPC, HPAEC, GC-MS, NMR analysis	Glucose, galacturonic acid	97 × 10^3^	Liu et al. (2020)
AC-ARPS	Distilled water	Phenol-sulfuric acid method, LC–MS, IR spectroscopy, ^1^H and^13^C NMR spectroscopy, HPGPC, AFM Congo Red experiment Pre-column derivatization, HPLC	Mannose, ribose, glucose, galactose, arabinose	25681	Yu et al. (2017)

### 2.1 Flavonoids and flavonoid glycosides

As plant secondary metabolites, flavonoids are main bio-active ingredients of AR. As shown in Figure 1, 28 flavonoids and flavonoid glycosides (compounds **1–28**) have been isolated. This group of glycosides mainly consists of 2-benzylchromone parent nuclei bearing C-3, C-6, C-7, C-8, C-3′, C-4′ and C-5′. The biological activity of flavonoids is related to their chemical structures. The antioxidant capacity is realized by terminating free radical chain reactions while binding to peroxyl radicals. Thong et al. found that by substituting the hydroxyl and methoxyl groups with two glucoses at 3 and 5′ sites of isorhamnetin significantly elevated its antioxidant capacity as compared to isorhamnetin (Thong et al., 2019).

In addition, the content of flavonoids varied along with producing areas and different varieties of AR (Shi et al., 2016). Among these flavonoids, the contents of quercetin, kaempferol, isorhamnetin-3-*O*-*β*-D-glucopyranoside and isorhamnetin are usually used as an index to evaluate the quality of AR (Liu et al., 2015).

### 2.2 Other glycosides

AR also contains other glycosides, such as kinsenoside, carotenoid, gastrodia (compounds **29–34**, Figure 2). Among these, kinsenoside is isolated from *n*-butanol site of methanol extract of AR, which is a glucoside formed by chiral carbon of 3(R)-hydroxy-γ-butanactone and β-D glucose connected by a glycoside bond (Wang et al., 2022). At present, kinsenoside is often used as a indicator for the quality control of AR, and has received much attention in studies of its biological activity such as hpyerglycemic, hypolipidemic, liver protection and anti-inflammatory activities (Lu et al., 2022). In addition, the research on kinsenoside is gradually transitioning to new drug clinical research (National Medical Products Administration, 2023), and these results indicate that the application of AR has achieved further success.

### 2.3 Polysaccharides

Polysaccharides extracted from AR have attracted considerable attention due to their promising potential in strengthening immunity, inhibiting tumor progression, as well as reducing body blood glucose and lipid content (Table 2).

Multiple chromatographic techniques were applied to excavate structural features of the polysaccharides in AR (ARPP). ARPP mainly comprises mannose, rhamnose, glucose, and galactose, while more than 75% are galactose and glucose. The bioactivity of polysaccharides is tightly correlated with its structure, including monosaccharide composition, ultrastructure, and spatial conformation. Researches isolated and purified four sub-fractions from the water-soluble polysaccharide ARPP-30, ARPP-60, ARPP-70a and ARPP-80. Results demonstrated that ARPP-80 showed most outstanding antioxidant capacity, followed by ARPP-70. Moreover, mannnose, arabinose and protein were high in ARPP80 (Zeng et al., 2016; Zhang Z. et al., 2021). Thus, it could be proposed that higher molecular weight and glucose content in ARPP might result in superior antioxidant activity.

### 2.4 Alkaloids

Despite the relatively low percentage of alkaloids in AR, they are still vital due to pharmacological activities, including anti-inflammatory, anti-bacterial, anti-virus, anti-cancer, and hepatocyte protection, etc. (Gong et al., 2014). Major alkaloids are huperzine A, aconitine, and anoectochine (compounds **35–37**, in Figure 3). However, theirs pharmacological activity has not been well studied. Thus, it’s of great necessity to dive deeper in this subject.

### 2.5 Other components

Apart from the above mentioned compounds, triterpenes, volatile oil, steroids, microelements, amino acids and nucleosides, have also been isolated from AR (compounds **38–76**, Figure 4). Triterpenoid saponins are vital components in edible and medicinal plants including *Atractylodes macrocephala* Koidz (Bailly, 2021) and ginseng (Chen et al., 2019), while most of them are pentacyclic triterpenes in AR. About 10 compounds have been identified, including *α*-incense resin, *β*-incense resin, friedelin, oleanolic acid, etc.

Various steroids and volatile oil were isolated, including campesterol, ergosterol, lanosterol, *β*-sitosterol, stigmasterol, palmitic acid, methyl hexacarboxylate, linolenic acid, etc. The volatile oil compounds can significantly increase myocardial activity and liver lipid secretion (Ye et al., 2017). Total amino acid, microelements found in AR are crucial in maintaining physiological, biochemical, and immune functions. Additionally, trace elements and amino acids take up larger proportion in AR as compared to American ginseng or wild ginseng, medicinal value is also increased (Shi et al., 2016).

## 3 Pharmacological effects

AR exerts various pharmacological effects, including anti-inflammation, anti-oxidation, anti-tumour and hypolipidemic. The pharmacological effects and potential mechanisms are summarized in Figure 5; Table 3.

**TABLE 3:** Summary of the pharmacological activities of AR extract/compounds.

Pharmacological activities	Extract/Compounds	Models (in vivo)	Results/Mechanisms	Dosages
Antidiabetic	Polysaccharides	Mice (STZ-induced)	GSH-Px↑ SOD↑ CAT↑ HDL-C↑ TG↓ LDL-C↓ MDA↓ TC↓	300 mg/kg
	Polysaccharides	Mice (high-fat diet and STZ-induced)	ALT↓ AST↓ TC↓ MDA↓ TG↓ LDL-C↓ SOD↑	100/300 mg/kg
	Kinsenoside	Mice (STZ-induced)	MDA↓ NO↓ NOS↓ Scavenging free radicals activity↑ SOD↑ iNOS↑	5/10/15 mg/kg
	Water extracts	Mice (alloxan-induced)	TG↑ LDL↑ HDL↑ SOD↑ GSH-Px↑ CAT ↑ MDA↑ V_C_↑ V_E_↑ TC↓	200/500 mg/kg
	Polysaccharides	T2DM mice (HFD and STZ-induced)	Ameliorates insulin sensitivity↑ HDL↑ treatment increases the β-cell area↑ hyperglycemia↓ G6Pase↓ LDL↓ triglyceride↓ PEPCK↓ cholesterol↓	1.5/3.0/6.0 mg/mL
	Polysaccharides	Mice (STZ-induced)	Hepatic glycogen↑ HDL-C↑ insulin levels↑ body weights↑ GSH-Px↑ CAT↑ SOD↑ LDL-C↓ TG↓ TC↓ blood glucose levels↓ MDA↓	50/100/200 mg/kg
	Polysaccharides	Mice (STZ-induced)	Body eights↑ glucose levels↓ FN↓ MCP-1↓ phos-MKK3/6↓ phos-p38↓TNF-α↓	100/300 mg/kg
Liver protection	Polysaccharides	Mice (CCl_4_-induced)	Liver index↓ ALT↓ AST↓	300 mg/kg
	Polysaccharides	Mice (CCl_4_-induced)	SOD↑ CAT↑ MDA↓ GSH-Px↑ GSH↑ liver index↓ ALT↓ AST↓	200/400 mg/kg
	Polysaccharides	Mice (CCl_4_-Induced)	ALT↑ AST↑ GSH↑ NOS↓ MDA↓ injured area↓ edematous liver cells↓ inflammatoryinfiltration↓	400 mg/kg
	Kinsenoside	Mice (17α-Ethinylestradiol-induced)	Weight↑ liver body index↑ FXR↑ BSEP↑ ALT↓ AST↓ TBA↓ BIL↓ IL-1β↓ IL-6↓ NTCP↓ ALP↓ the liver pathological-symp-toms↓ CYP7A1↓ extensive expression of CK19↓	50/100/200 mg/kg
	Kinsenoside	Mice (CCl_4_-induced)	CYP2E1↑ ALDH↑ AMPK↑ TG↓ AST↓ body weight ratio↓ ALT↓ ADH↓ ADH1B↓ the lipid accumulation levels↓	20/40 mg/kg
	Kinsenoside	Mice (MCD/HFFD-induced)	Lipid accumulation↓ inflammation↓ fibrosis↓ AST↓ ALT↓ SOD↓ MDA↓ IL-12↓ p70↓ IL-6↓ TNF-α↓ MCP-1↓ IFN-γ↓ α-SMA↓ Col-I↓ TIMP-1↓	10/20/40 mg/kg
	Kinsenoside	Mice (Radiation-induced)	α-SMA↓ RILF↓ TGF-β1↓ fibrosis↓ TGF-β1/Smad/CTGF↓	20 mg/kg
	Quercetin	Mice (CCl_4_-induced)	Improve the histopatho-logical, p-IĸBa↓ ALT↓ NF-kBp65↓ AST↓	60/120 mg/kg
	Polysaccharides	Mice (CCl_4_-induced)	SOD↑ CAT↑ GSH-Px↑ GSH↑ liver index↓ ALT↓ AST↓ NF-κBp65↓ KC↓ inflammatory cells infiltration↓ TGF-β1↓ MDA↓ CYP2E1↓ TNF-α↓ IL-6↓ MCP-1↓ MIP-2↓	150/300/500 mg/kg
	Water extracts	Mice (CCl_4_-induced)	T-AOC↑ GSH↑ AST↓ ALT↓ TG↓ MDA↓ SOD↑ inflammation↓ acuolar degeneration↓	75/150/300/500 mg/kg
	Polysaccharides	Mice (HFD-induced)	Liver weight↓ insulin sensitivity↑ PGC1α↑ Nrf2↑ NQO1↑ HO-1↑ TNF-α↓ IL-6↓ HDL↑ LDL↓ TC↓ ROS↓ inflammatory responses↓ bnormal lipid metabolism↓ blood lipid↓	100/200/400 mg/kg
	Polysaccharides	Mice (HFD-induced)	Cognitive function↑ learning and memory↓ glucose and lipid metabolic disorders↓ neuroinflammation↓ inflammation in colon↓ ameliorate impaired intestinal barrier, reshaped the gut microbiome	1/3 mg/g
Anti-inflammatory	Polysaccharides	Mice (MSU-induced)	Protects HUVECs Prevents, the impairment of HUVECs NF-κB↓ JNK/Erk↓ attenuated MSU uptake in↓ inflammatory penetration↓ protective efficacy of endothelial cell, ttenuation of MSU-induced ankle swelling tail-flick response and writhing reaction	
	Kinsenoside	Mice (type II collagen -induced arthritis)	IL-10↑ arthritis index↓ IL-1β ↓ IL-6↓ TNF-α↓ inflammation↓destruction of joints↓ inflammation↓ NF-κB↓ destruction of joints↓ p-p65↓ synovial hyperplasia↓	30/120 mg/kg
Antioxidant	Water extracts	Mice (alloxan-induced)	HDL↑ SOD↑ CAT↑ GSH-Px↑ MDA↑ blood glucose levels↓ TG↓ TC↓	0.5/2.0 g/kg
	Flavonoids	Mice (H_2_O_2_/D-gal-induced)	SOD↑ GPx-1↑ GPx-4↑ MAO↑ GSH-PX↑ MDA↓	122.5/245/490 μg/mL
	Phenols	Mice (D-galactose-induced)	ABTS free radical↓ MDA↑ T-SOD↓ GSH-Px↓	50/100/200 mg/kg
Angioprotective	Polysaccharide	Mice (HFD/STZ-induced)	TIMP-1↑ TIMP-2↑ collagen deposition in the vessel wall↓ p-NF-κB p65↓ p-p38MAPK↓	100/300 mg/kg
	Kinsenoside	Mice (STZ-induced)	SOD↑ CAT↑ TIMP-1/-2mRNA↑ NO↑ NF-κB↓ MMP-2/-9↓	20/50/100 mg/kg
	Kinsenoside	Mice (H_2_O_2_-induced)	Attenuates RPE cell apoptosis, protect RPE cells inhibits VEGF release, inhibits neovascularization	1600/3200 µM

### 3.1 Antidiabetic activities

Diabetes is one of the most dangerous non-communicable diseases, and it is ranked as the fourth leading cause of death worldwide. Plant-derived natural remedies with hypoglycemic action are gaining increasing popularity, among which AR is a promising curing agent (Ye et al., 2017).

The studies showed that extracts of AR exhibited potent anti-diabetic effects, which is related to polysaccharides. The mechanism is possibly related to the antihyperglycemic, antioxidant and antihyperlipidemic activities. Tang et al. (2018) studies have found that AR polysaccharide could alleviate STZ-induced diabetic mice by reducing the oxidative stress response (ROS, MDA, SOD, CAT, GSH-Px). Liu et al. research finding the vascular protective effects of ARP might be associated with NF-κB and p38 MAPK pathway. And AR polysaccharide might be used as useful substance in the treatment of vasculopathy in diabetic patients. Furthermore, the administration of ARPs-p can mediate antioxidant activity to protect the pancreatic islets from free radical damage and ameliorate the hyperglycemic and oxidative stress and hypolipidemic properties in STZ-induced diabetic mice; this indicates that ARPs-p possesses dramatic antidiabetic activities. These results firstly revealed the effects of the main active component of ARPs-p (i.e., β-(1 → 3)-D-glucan) on antidiabetic activities and provided a valuable theoretical basis for the investigation of the structure–function relationships of polysaccharides (Tang et al., 2018; Liu et al., 2017; Liu et al., 2020). Another study showed that polysaccharides significantly downregulated hyperglycemia in C57BL/6J mice with STZ-induced type 2 diabetes, mainly by increasing insulin sensitivity, inhibiting hepatic gluconeogenesis, and reducing serum triglyceride, cholesterol, and LDL levels, while at the same time, doing negligible harm to major organs (Gao H. et al., 2021).

These findings suggest that AR is a promising candidate for the prevention and treatment of diabetes. Although several studies have demonstrated the definite hypoglycemic effect of ARPP, more investigations are required to identify the specific bioactive-structural features responsible for this activity.

### 3.2 Hepatoprotective activity

Polysaccharides and kinsenoside isolated from AR were found to be promising in the treatment of chronic and acute hepatitis by constructing liver injury models. The liver protection mechanisms are anti-oxidative stress, reducing inflammation, liver fibrosis, etc.

Gao L. et al. (2021) found that kinsenoside could alleviate alcoholic liver injury by inhibiting oxidative stress and ER stress while activating AMPK-dependent autophagy. In CCl_4_-induced hepatic damage model, Yu et al. (2021) demonstrated the protective effect of polysaccharides on liver function in SD rats with acute liver injury, this could be explained by the attenuation of oxidative stress, inflammation, and apoptosis (Zeng et al., 2017). At the same time, researchers figured out that polysaccharides regulate amino acid metabolism, lipid metabolism, gut bacteria metabolism, methylation, and energy metabolism, in order to ameliorate the abnormalities caused by CCl_4_ (Zeng, 2020; Zeng et al., 2020). Besides, the cholestatic model was constructed to further support the above conclusion; results demonstrated that kinsenoside could alleviate liver pathologic damage, serum biochemical status, inhibit hepatocellular microstructure disorder and bile duct hyperplasia, by inhibiting inflammatory responses and regulating bile acid homeostasis (Ming et al., 2021). In the concanavalin A-induced T cell-mediated hepatitis SD rat model, kinsenoside could elicit immunosuppression against autoimmune liver injury by targeting VEGFR2, followed by diminishing the cross-talk of metabolism-related PI3K-AKT and inflammation-related JAK2-STAT3 pathways (Xiang et al., 2016).

Another study showed that kinsenoside remarkably reduced lipid accumulation, inflammation, and fibrosis in the NASH mice liver. This was realized by inhibiting NF-κB/NLRP3 signaling pathway (Deng et al., 2022); in addition, both *in vitro* and *in vivo* experiments discovered that kinsenoside could inhibit the radiation-induced liver fibrosis by regulating CTGF and TGF-β1/Smad/CTGF pathway; moreover, kinsenoside exhibited negligible adverse effects during tumor radiotherapy. These results have provided supporting evidence for the clinical application of kinsenoside as an innovative drug for the treatment of the liver fibrosis (Nie et al., 2022).

Furthermore, ARPP80 not only has the effect of protecting liver, its mechanism may be related to antioxidant, but also has a strong hepatoprotective effect of ARPP80, which is comparable to silymarin, and should be explored as a new natural supplement (Xu et al., 2017). Collectively, AR is promising in accelerating the development of a dietary supplement or medication to improve liver function. Yet, further investigations on its effective monomer compounds, molecular mechanism, and even clinical studies are required.

### 3.3 Anti-inflammatory

Polysaccharides and kinsenoside showed anti-inflammatory properties. Researchers suggested that polysaccharides was promising in curing type II collagen induced arthritis, which was realized by down-regulating TNF-α, IL-1β and IL-6 and up-regulating IL-10 expression; the possible mechanisms could be the inbition of the NF-κB pathways (Guo et al., 2019). Another study showed that kinsenoside had an obvious therapeutic effect on monosodium urate (MSU)-induced gouty arthritis; the results indicated that kinsenoside suppressed the MSU-stimulated proliferation and induce apoptosis induction of HUVECs cell via targeting IKKα and IKKβ kinases in macrophages. This further suppress NF-κB signaling and related cytokine expressions and subsequent endothelium bioactivity (Han et al., 2016). These studies have confirmed the potential of AR to reduce of cellular inflammatory cytokines, apoptosis, and inhibition of the NF-κB signaling pathway to relieve inflammation, but the other underlying mechanisms remain unclear.

### 3.4 Anti-tumour activity

Cancer is a leading cause of death worldwide, accounting for nearly ten million deaths in 2020 (Sung et al., 2021). TCM has become an indispensable therapeutic strategy (Mao et al., 2022), while AR has long been used for treating human colon cancer, esophageal cancer OE19. Zhang et al. figured out that petroleum extract of AR exerted a strong inhibitory effect on human colon cancer, with an IC_50_ value of 45.51 ± 1.66 μg/mL (Zhang et al., 2017). Another study found that under the concentration of 5.67 ± 0.831 μmol/L, polysaccharide could significantly inhibit the proliferation of esophageal cancer OE19 by inducing apoptosis and G2/M phase arrest (Yu et al., 2017). Furthermore, it has been proved that the essential oil components in AR could inhibit the proliferation of small cell lung cancer cells by activating apoptosis (Chen et al., 2012).

These study provides novel directions for developing new drugs from AR. However, the restricted permeability and low bioavailability have hampered its clinical application. Nano-drug delivery system (NDDS), such as liposomes, offer a promising way to enhance the anti-cancer effect of traditional Chinese herbs (Fu et al., 2022).

### 3.5 Antioxidant activity

Oxidative stress and the generation of free radical have been linked to various chronic diseases (Martemucci et al., 2022). Several studies reported that flavonoids, polysaccharides, kinsenoside and phenols in AR with antioxidant effects could. protect LO2 cells against H_2_O_2_ induced oxidative stress and exerted potent anti-aging effects in D-galactose aging mouse model (Wang H. G. et al., 2020). Besides, ARPPs was reproted with antioxidant and hepatoprotective capacity against CCl_4_ injection; the underlying mechanisms the hepatoprotective effects of ARPPs might, therefore, involve attenuating oxidative stress. Moreover, the total phenolics extraction showed significant antioxidant activity by significantly decreased MDA content, while activating T-SOD and GSH-Px activities (Xu et al., 2017).

Luo et al. (2018) found that kinsenoside protected retinal pigment epithelium from apoptosis against H_2_O_2_-induced oxidative injury, while simultaneously decreasing apoptosis-related neovascularization; this phenomenon could be explained by the inhibition of Erk/p38/NF-κB signaling pathway. Moreover, kinsenoside ameliorated mitochondrial dysfunction and enhanced antioxidant capacity through the Akt/Nrf2/HO-1 signaling pathway in myocardial ischemia-reperfusion injury (Wang et al., 2023).

In conclusion, the underlying mechanism of the antioxidant activity of AR is mainly through regulation of signaling pathways such as Erk/p38/NF-κB, Akt/Nrf2/HO-1 and AMPK/Nrf2, etc. The favorable effects of AR on alleviating inflammatory and oxidative damages have been confirmed, but the practical application is a double-edged sword as gap between effective dose and toxic dose *in vivo* is unknown. The antioxidant activity of AR has great potential and should be studied in more depth.

### 3.6 Immunomodulatory activity

The immune system is vital in guarding human body from the invasion of disease-causing substances. Several polysaccharides have been systematically investigated for their immunomodulatory effects (Ye et al., 2017). Polysaccharide could improve immune activity by promoting the proliferation of splenocytes and secretion of NO as well as cytokines of IL-2, IL-6, and IFN-γ (Ma et al., 2018). AR can be considered an effective herbal medicinal product for modulating the immune system. Notably, about the relationship between immunomodulatory effects and structure of polysaccharide is still lack.

## 4 Quality control

Both the global production and application of AR are increasing tremendously. Thus, it's essential to construct a comprehensive and general standard for AR quality evaluation. Currently, the local standards for food safety in Fujian Province (DBS 35/006-2022, 2022) require that kinsenoside in AR (fresh or anhydrous substance) should be more than 10% to ensure medicinal quality (Fujian Provincial Health Commission, 2022). However, quality control is still inadequate both in the Fujian and Anhui Provincial Standard of Chinese Medicinal Materials. Which resulted in varied quality on market. This seriously affects the safety and the efficacy of TCM prescriptions, not to mention the development question.

The quality of AR related with multiple factors including germplasm, producing area, harvesting, processing, preparation, and storage. These factors greatly influence the content of compunds. A high-quality strain is a crucial assurance of medicinal material quality and standardization of Chinese medicinal materials; except for character identification and microscopic identification, Wang L. et al. (2020) used a new method of SSR molecular markers for classification and identification; it is widely used in the construction of genetic linkage map for the preservation of genetic resources. In addition, the quality of AR varied in species and production areas is different; Chen et al. (2021) figured out that methanol extracts content were significantly among 10 producing areas and 5 strains, and the content of kinsenoside were affected by the plantation period; in another study, Zhang et al. (2020) reported that the content of five flavonoids (rutin, narcissoside, quercetin, kaempferol and isorhamnetin) also differed significantly among 4 strains. Besides, drying is the traditional processing method for AR, however, the contents of 8 flavonoids (rutin, isoquercitrin, kaemprerol-3-*O*-rutse, narcissoside, quercirein, quercetin, kaempferol and isorhamnetin), 6 nucleosides (cytidine, uridine, adenine, guanosine, *β*-thymidine and adenoside), kinsenoside and total flavonoid were affected by the different dried temperatures (Zhang X. et al., 2022). Thus, a single index is hardly adequate to meet the needs of the quality control. The quality evaluation of AR should consider all elements, including strains, planting, production, and processing.

The active components of AR are analyzed using analysis methods such as ultra-high performance liquid chromatography-multistage mass spectrometry (UPLC-MSn), ultraviolet spectrophotometry, HPLC, ect. In recent years, the concept of quality markers (Q-markers) of traditional Chinese medicine was developed, which could reflect the relationship between the quality of TCM components and the curative effect (Liu et al., 2016). The quality markers of traditional Chinese medicine referred to chemical substances inherent in Chinese medicinal materials or formed in the process of processing and preparation that are closely related to the functional attributes of traditional Chinese medicine. They are the landmark substances reflecting the safety and effectiveness of traditional Chinese medicine. Q-marker-based predictive analysis showed wider application in quality control of traditional Chinese medicine (Guan et al., 2022). Future research could focus on predicting and analyzing the Q-markers of AR from the perspectives of plant kinship, efficacy, drug properties and measurability of chemical components, to establish safer, more scientific and effective quality evaluation methods.

## 5 Applications

**FIGURE 6:**
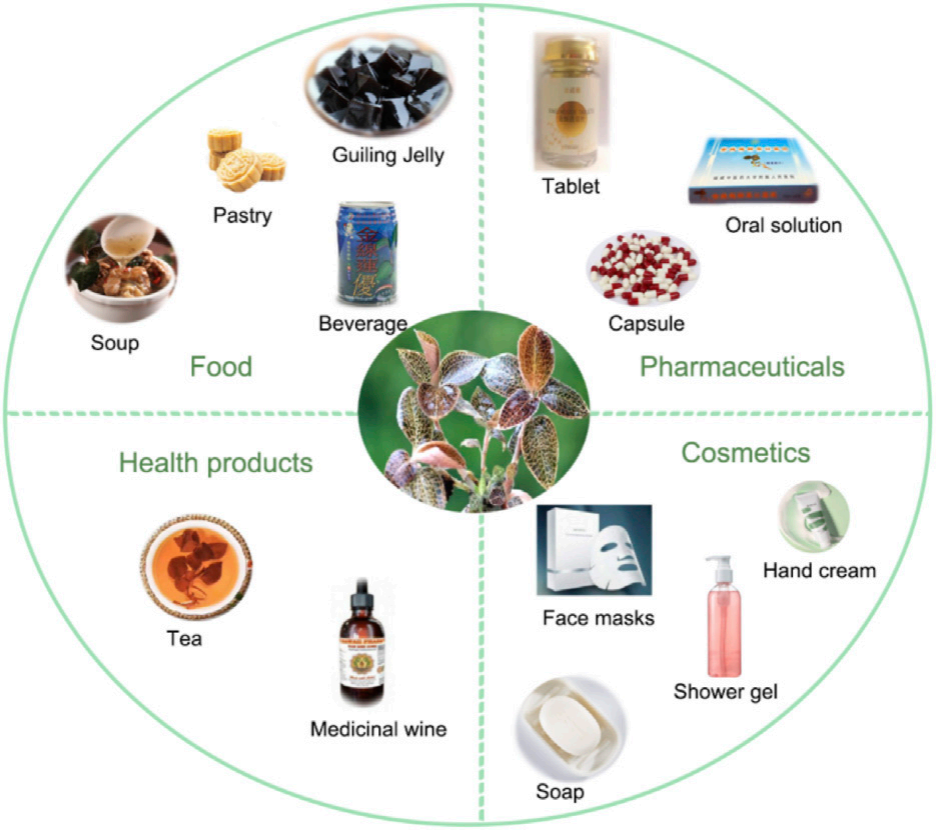
The applications of AR in pharmaceutical, food, health products, and cosmetics.

Apart from the outstanding medicinal value, AR is also promising in the fields of healthcare products, food and cosmetics (Figure 6; Table 4).

**TABLE 4:** The patents for AR.

NO.	Patent name	Approval number
Pharmaceutical		
1	Anoectochilus roxburghii compound hypoglycemic oral solution	CN 115154553 A
2	Kinsenoside is used for the preparation of drugs for radioactive liver fibrosis	CN 113730423 A
3	The use of Kinsenoside in the preparation of drugs against neuroinflammation	CN 110585227 B
4	Use of Kinsenoside in the preparation of drugs to remove β amyloid plaques in the brain	CN 110585225 B
5	Anoectochilus roxburghii enzyme with improving immunity and its preparation method	CN 113230353 A
6	Application of Anoectochilus roxburghii in preparing drugs for the prevention and/or treatment of pulmonary fibrosis	CN 112870212 A
7	A method for preparing fresh juice and oral liquid, capsules, and granules of Anoectochilus roxburghii	CN 102150855 A
Food		
1	Anoectochilus roxburghii with passion fruit and lemon tea	CN 116849285 A
2	Anoectochilus roxburghii pastry and its preparation method	CN 115568485 A
3	Anoectochilus roxburghii Guiling jelly and its preparation method	CN 109170884 A
4	Anoectochilus roxburghii beverage and its preparation method	CN 115486506 A
5	Anoectochilus roxburghii atis craft beer and its preparation method	CN 116656441 A
Health products		
1	A method of making Anoectochilus roxburghii health wine	CN 109706047 A
2	A health Anoectochilus roxburghii tea tablet and its preparation method	CN 115644286 A
Cosmetic		
1	A separation and purification method of Anoectochilus roxburghii and its application in soothing cosmetics	CN 114634535 A
2	Nine Grass Green Cream with Whitening and Spot Removing Effects and its preparation method	CN 104434618 A

As mentioned before, AR contains numerous active compounds for pharmaceutical applicationsincluding the prevention and treatment of radioactive liver fibrosis (Zhang Y. H. et al., 2021). Furthermore, AR is also used in the field of health products, such as health wine and tea (Wang et al., 2019; Xu et al., 2022). Additionally, it is also widely used in the food industry, such as Guiling jelley and pastry (Zeng, 2023; Weng, 2018), which offers a wide range of dietery applications.

Besides, AR extract is also used in facial mask, soap and shower gel cosmetics, with beneficial characteristics with anti-aging, whitening, reducing skin wrinkles and moisturizing (Liu et al., 2023). Therefore, with the increasing research efforts, its potential for further commercial application in the cosmetics and food industry should be explored.

## 6 Conclusion and perspectives

The whole plant of AR has long been used in the treatment of clearing heat and cooling blood, dispelling wind, removing wind and dampness, balance Yin and Yang, which was recorded in “Compendium of Materia Medica.”The traditional medicinal efficacies of AR are treatments of tuberculosis hemoptysis, diabetes, nephritis, cystitis, myasthenia gravis, rheumatism and rheumatoid arthritis, febrile convulsion and snakebite (“National Compendium of Chinese Herbal Medicine” Editorial Board, 1978; Chinese Herbalism Editorial Board, 1999; Jiangsu Institute of Botany, 1990). In modern treatment, the plant is a promising source for curing diabetic, liver-injury and tumor. Based on the progress in phytochemistry, the pharmacological activities of these plant-derived compounds were systematically investigated and bioactive components were screened out. The main active ingredients of clematis are quercetin, ishamhamin, lotus, polysaccharide, etc., which have anti-diabetes, hepatoprotection, anti-inflammatory and other pharmacological activities, and are used for the treatment of diabetes, liver injury, inflammation and other diseases.

These findings expand the clinical application of AR. However, the potential mechanisms underlying the pharmacological activities remain to be elucidated. Also, it’s crucial to investigate the internal relationship between the chemical composition and pharmacological activities. Pharmacokinetic studies should also performed to clarify the absorption, distribution, metabolism, excretion and predict the potency and toxicity of these bio-active compounds. Moreover, it’s of great necessity to establish a comprehensive set of quality standards to distinguish different AR, as the content of chemical compositions differed among cultivation, tissue culture, or even different strains of AR.

By far, the chemical compositions of AR have been well-investigated, with approximately 76 structures are identified. Among them, flavonoids and polysaccharides represent the main ingredients, while other chemical constituents like alkaloids, lignans and amino acids are few. Thus, more chemical constituents should be derived to thoroughly investigate SAR.

Although AR has good activity and can be applied in many aspects, due to the lack of quality control standards, the quality of commercial clematis is mixed, resulting in the consequences of the advantages and disadvantages and the poor effect of medicine, this seriously affects the safety and the efficacy of TCM prescriptions, not to mention the development question, so quercetin, kaempferol, concematis and other indicators can be used as the evaluation criteria. In this way, Q-markers method is adopted for the discovery of chemical compositions and quality control, in order to establish a more comprehensive and safer quality evaluation.

To sum up, AR exerts multiple functions as medicine, food, and cosmetics, which has been widely used as whitening or healthcare product. Nevertheless, converting medicinal herbs into food and cosmetics products is a major trends in recent years. Further investigation in these fields may have broader prospects for future development of AR.
